# C-Reactive Protein (CRP) and Autoimmune Disease: Facts and Conjectures

**DOI:** 10.1080/17402520400001751

**Published:** 2004

**Authors:** Alexander J. Szalai

**Affiliations:** Division of Clinical Immunology and Rheumatology Department of Medicine The University of Alabama at Birmingham Birmingham AL USA

## Abstract

C-reactive protein (CRP) is a blood component comprised of five identical subunits with a combined molecular mass of 110 kDa; in the presence of Ca^++^ it binds phosphocholine (PC) with high affinity. Ligand-bound CRP activates complement and the protein reportedly binds various Fc receptors. Coincident with a now decade-long resurgence in clinical interest in associations of CRP with disease, our laboratory has been investigating the biology of CRP *in vivo* using human CRP transgenic mice (CRPtg). At that time we confirmed that CRP affects a host defense function mediated at least in part through the elimination of pathogens. Less appreciated and not as well understood as CRP's ability to bind antigen and aid in the elimination of microbes, is its known ability to bind autoantigens and presumed capacity to promote clearance of apoptotic cells. These latter properties of CRP have long been suspected to contribute to homeostasis and to autoimmune disease. In this article we review and update the evidence generated in CRPtg by our group and *in vitro* by others' that indicates CRP is more than just an antimicrobial molecule and convenient marker of inflammation - rather, it protects against autoimmunity. A mechanistic hypothesis is presented to account for this cause-and-effect relationship.


*Clinical & Developmental Immunology*, September/December 2004, Vol. 11 (3/4), pp. 221–226

Taylor & Francis healthsciences

### C-Reactive Protein (CRP) and Autoimmune Disease: Facts and Conjectures

ALEXANDER J. SZALAI*

*Division of Clinical Immunology and Rheumatology, Department of Medicine, The University of Alabama at Birmingham, 437B Tinsley Harrison Tower, 1530 3rd Avenue South, Birmingham, AL 35294-0006 USA*

C-reactive protein (CRP) is a blood component comprised of five identical subunits with a combined molecular mass of 110 kDa; in the presence of $Ca^{++}$ it binds phosphocholine (PC) with high affinity. Ligand-bound CRP activates complement and the protein reportedly binds various Fc receptors. Coincident with a now decade-long resurgence in clinical interest in associations of CRP with disease, our laboratory has been investigating the biology of CRP *in vivo* using human CRP transgenic mice (CRPtg). At that time we confirmed that CRP affects a host defense function mediated at least in part through the elimination of pathogens. Less appreciated and not as well understood as CRP's ability to bind antigen and aid in the elimination of microbes, is its known ability to bind autoantigens and presumed capacity to promote clearance of apoptotic cells. These latter properties of CRP have long been suspected to contribute to homeostasis and to autoimmune disease. In this article we review and update the evidence generated in CRPtg by our group and *in vitro* by others' that indicates CRP is more than just an antimicrobial molecule and convenient marker of inflammation—rather, it protects against autoimmunity. A mechanistic hypothesis is presented to account for this cause-and-effect relationship.

*Keywords*: SLE; MS; Inflammation; Lupus; EAE; Apoptosis

### WHAT IS CRP?

C-reactive protein (CRP) is one of only two acute phase proteins known as "pentraxins" (Osmand *et al.*, 1977; Gerwurz *et al.*, 1995). They share extensive sequence and structural homology (Emsley *et al.*, 1994; Gerwurz *et al.*, 1995; Shrive *et al.*, 1996) and are differentiated based on function; those that bind best to phosphocholine (PC) are CRP-like (Volanakis and Kaplan, 1971; Anderson *et al.*, 1978) while those that bind best to phosphoethanolamine (PE) are serum amyloid P-component (SAP)-like (Hind *et al.*, 1985; Schwalbe *et al.*, 1992). Most animals make both but usually only one is an acute phase reactant (Kushner and Mackiewicz, 1993), thus in humans within hours after an inflammatory insult, blood CRP level rises dramatically but SAP does not (Kushner, 1982; Pepys and Baltz, 1983; Kushner and Mackiewicz, 1993), whereas in mice SAP increases with kinetics and magnitude like CRP in humans (Pepys *et al.*, 1979). Unique among mammals, mouse CRP is always a trace blood component (Bodmer and Siboo, 1977; Whitehead *et al.*, 1990).

Because of its predictable acute phase behavior in humans, measurement of blood CRP has proven clinical utility, and extensive data has been collected showing significant statistical association between CRP level and likelihood and severity of various inflammatory diseases, including autoimmunity (Young *et al.*, 1991; Morrow and Ridker, 2000). Here we review and update evidence generated by our group and others' that indicates CRP is more than just a convenient marker of inflammation—rather, the association of CRP with autoimmune disease arises from underlying biology, i.e. cause-and-effect relationships. A hypothesis is then proffered to account for these relationships.

### WHAT DOES CRP DO AND HOW DOES IT DO IT?

The first activity ascribed to CRP was its ability to precipitate the *Streptococcus pneumoniae* cell wall teichoic acid, C-polysaccharide (Abernathy and Avery, 1941). The reaction depends on the protein's $Ca^{++}$ dependent binding to PC (Volanakis and Kaplan, 1971), a major constituent of at least two cell wall antigens common to all serotypes of pneumococci; teichoic acid and lipoteichoic acid (Tomasz, 1967; Fischer, 2000). PC was thus the first CRP ligand discovered and so far is the best characterized. Unfortunately, the fact that CRP binds to ligands other than PC is often overlooked. Among the lesser appreciated CRP ligands are a variety of

*Corresponding author. Tel.: +1-205-975-6241. Fax: +1-205-934-1564.

ISSN 1740-2522 print/ISSN 1740-2530 online © 2004 Taylor & Francis Ltd
DOI: 10.1080/17402520400001751

autoantigens including chromatin, histones, U1 small nuclear ribonucleoprotein, nuclear envelope protein, and apoptotic cells (Robey *et al.*, 1985; Du Clos *et al.*, 1988; Du Clos, 1989; Minota *et al.*, 1993; Gershov *et al.*, 2000; Lee *et al.*, 2002). Binding to most of these ligands requires $Ca^{++}$ and can be inhibited by PC, indicating that a single promiscuous ligand-binding site on CRP accommodates diverse structural groups. Details about the architecture of the ligand-binding site emerged from protein mutagenesis studies (Agrawal *et al.*, 1992; 1997) and solution of the crystal structure of native CRP (Shrive *et al.*, 1996). CRP *per se* consists of five non-covalently associated subunits of 206 amino acids arranged in cyclic symmetry. Each subunit has one PC-binding site, and all five binding sites are oriented towards the same face of the pentamer. As a result, CRP can associate with high avidity to surfaces that display appropriate ligands in an array, such as on the surface of cells undergoing apoptosis.

Purified CRP bound to a target can initiate and regulate complement activation. CRP activates the classical pathway (CP) and reduces alternative pathway (AP) generation of the cell-lytic C5b-9 complex, and the protein inhibits lectin pathway associated deposition of the C3b opsonin (reviewed in Mold *et al.*, 1999). Insofar as is known CRP's complement activating effect relies on binding to the protein C1q, and its inhibitory effect depends on binding factor H. At least *in vitro*, complement activation by ligand-bound CRP: (1) promotes the CP mediated deposition of C3b and C4b onto CRP/ligand complexes, (2) mediates solubilization of antigenic CRP/ligand complexes, and (3) supports subsequent recognition of the complexes by complement receptors on phagocytes (Volanakis, 1982a,b; Volanakis and Narkates, 1983; Mold *et al.*, 1996; 1999). Complement activation by CRP reportedly does not support formation of an effective C5-convertase (Berman *et al.*, 1986).

Other studies have shown that purified human CRP binds FcγR expressed by both human and mouse cells (Marnell *et al.*, 1995; Bharadwaj *et al.*, 1999; Stein *et al.*, 2000; Mold *et al.*, 2001). X-ray crystallography revealed a cleft on each CRP subunit, directly opposite the ligand-binding site, which presumably accommodates binding of both C1q and FcγR (Agrawal and Volanakis, 1994; Agrawal *et al.*, 2001). Notwithstanding the good evidence showing CRP/FcγR interaction, this remains a somewhat controversial issue because a few investigators have concluded that CRP is not bound by leukocyte FcγR at all (for example Hundt *et al.*, 2001). How might the single promiscuous binding site differentiate between interactions with C1q versus FcγR, and how might the seemingly disparate *in vitro* findings be resolved? The answers remain equivocal but both questions are actively being pursued by our group; we believe the recent discovery that human CRP sometimes exists as a glycoprotein (Das *et al.*, 2003) might offer a potential resolution. Briefly, Mandal's group showed that acute phase CRP isolated from patients with inflammatory conditions, including systemic lupus erythematosus (SLE), was glycosylated. This was unexpected because human CRP had previously been thought to be unglycosylated. Glycosylation of CRP is a post-translational event and importantly, carbohydrate attaches to the floor of the protein's C1q/FcγR binding cleft (Das *et al.*, 2003). Based on the known effect that glycosylation has on the ability of immunoglobulins to bind FcγR (Jefferis and Lund, 2002) and to activate complement (Wright and Morrison, 1997), we therefore predict that the C1q and FcγR binding capacities of CRP are controlled by its glycosylation state. If further study confirms this conjecture, this might explain some of the seemingly paradoxical FcγR-binding results.

### ADMINISTERED AND TRANSGENIC HUMAN CRP PROTECTS LUPUS-PRONE MICE

Regardless of the exact nature of the interaction, it is reasonable to suppose that CRP binding to apoptotic cells, coupled with its ability to recruit complement and FcγR mediated effector pathways, could modify the disease phenotype in autoimmunity. In fact already there is strong clinical and experimental evidence implicating involvement of CRP in the pathogenesis of SLE. In most diseases the blood level of CRP accurately reflects the degree of underlying inflammation and tissue necrosis (Ballou and Kushner, 1992), yet in SLE patients there is often little or no increase in CRP (Pereira *et al.*, 1980; Pepys *et al.*, 1982; Gabay *et al.*, 1993). As already mentioned, CRP binds to autoantigens of relevance in SLE (Robey *et al.*, 1985; Du Clos *et al.*, 1988; Du Clos, 1989; Minota *et al.*, 1993; Gershov *et al.*, 2000), and ligand-complexed CRP can recruit the complement system and Fcγ receptors in both mouse and man. Thus via deposition of complement cleavage fragments on both CRP and on the ligand (Volanakis, 1982c; Volanakis and Narkates, 1983) CRP can mediate complement-dependant solubilization of chromatin (Robey *et al.*, 1985) and likely assists in the clearance of nuclear material from the circulation by recruiting complement and FcγR. Early evidence that this actually occurs *in vivo* is the detection of CRP in immune complexes purified from sera of SLE patients, and reports that human CRP administered to mice effects plasma clearance of nucleosome core-particles and chromatin (Du Clos *et al.*, 1994; Burlingame *et al.*, 1996).

Among mice the NZB/NZW hybrid strain (i.e. BW mice) most accurately reflects the disease process involved in human SLE; they display a pathognomonic anti-nuclear autoantibody response that includes anti-double stranded DNA (dsDNA) and they spontaneously develop fulminate autoimmune glomerulonephritis (Theofilopoulos and Dixon, 1989). Also like humans the disease is most frequent in females, susceptibility is influenced by the MHC (mouse *H-2*), and non-*H-2* loci are linked to the organ-specific symptoms (Theofilopoulos and Dixon, 1989; Vyse and Kotzin, 1998). Like the aberrant expression of CRP in SLE patients, BW mice have an impaired SAP acute phase response (Rekvig *et al.*, 1998). This is noteworthy as like CRP, SAP binds chromatin and certain $SAP^{-/-}$ mice spontaneously develop autoimmune nephritis (Bickerstaff *et al.*, 1999). Injected human CRP had been shown to offer some protection against lupus in BW mice immunized with chromatin (Du Clos *et al.*, 1994), but other than a reported transient decrease in anti-DNA and anti-histone antibodies after the initial injection of chromatin, infused CRP did not affect the autoimmune response and the beneficial effect was short-lived. The transient nature of the CRP protective effect was attributed to the development of an anti-CRP response in mice receiving human CRP.

Using a human transgene Rüther made CRP transgenic mice (CRPtg) (Ciliberto *et al.*, 1987) that express human CRP as an acute phase protein and achieve blood levels of CRP comparable to those in humans. We have since generated many modified variants of Rüther's original CRPtg, and have used these to extensively study CRP's biological properties *in vivo*. Importantly, in CRPtg generation of anti-human CRP is not a concern (Klein *et al.*, 1998). We recently showed that lupus-prone BW mice carrying the CRP transgene had reduced proteinuria and lived longer than non-transgenic BW (Szalai *et al.*, 2003). They also had lower IgM and higher IgG anti-dsDNA titers than BW, accumulation of IgM and IgG in the renal glomeruli was delayed, reduced, and more mesangial than in BW, and end-stage accumulation of IgG, IgM, and C3 in the renal cortex was prevented. Like in some SLE patients, with disease progression in CRPtg/BW there was little rise in plasma CRP; however, to our surprise the protein became increasingly apparent in the kidneys due to local expression of the transgene.

Our observation that IgM anti-dsDNA is lowered in CRPtg/BW mice is consistent with a model recently proposed to explain the autoimmunity preventing properties of SAP (Bickerstaff *et al.*, 1999). Accordingly, CRP might be preventing activation of autoreactive B cells by promoting clearance of autoantigens (i.e. those displayed by apoptotic cells) to non-antigen presenting sites in the liver, and/or CRP may target delivery of autoantigens to the bone marrow where autoreactive B cells are then deleted or anergized. These possibilities have not yet been investigated.

### TRANSGENIC HUMAN CRP IS PROTECTIVE IN A MOUSE MODEL OF MULTIPLE SCLEROSIS

Like lupus, we showed that CRPtg mice resist experimental autoimmune encephalomyelitis (EAE), an animal model of multiple sclerosis (MS) (Szalai *et al.*, 2002). In CRPtg compared to wildtype, the duration of the human CRP acute phase response accompanying the inductive phase of EAE correlated with a delay in disease onset. In fact, there was little or no infiltration of the spinal cord by $CD3^+$ and $CD11b^+$ inflammatory cells and EAE was sometimes prevented altogether. CRPtg also resisted EAE induced passively via transfer of encephalitogenic T cells from symptomatic wildtype donors. Human CRP had three effects on cultured encephalitogenic cells that likely contributes to the protective effect we observed: (1) CRP inhibited encephalitogenic peptide-induced proliferation of T cells; (2) CRP inhibited production of inflammatory cytokines and chemokines (TNF-α, INF-γ, MIP-1α, RANTES, MCP-1); and (3) CRP increased IL-10 production. Unlike in CRPtg/BW all three of these actions were realized only in the presence of high concentrations of human CRP. Ongoing studies have revealed that CRP still mediates resistance to EAE in CRPtg that lack a functional complement system, but CRPtg lacking certain FcγR are not protected at all. In their entirety these data suggest that during the acute-phase of inflammation in CRPtg with EAE, the high level of circulating human CRP achieved acts (in)directly on an FcγR-expressing cell(s) and thus inhibits the damaging action of inflammatory cells and/or T cells that otherwise support onset and development of EAE.

### CRPtg MICE RESIST EXPERIMENTALLY-INDUCED APOPTOSIS

Lethal apoptosis can predictably be caused in mice by injection of TNF-α or certain agonistic anti-Fas antibodies (Xu *et al.*, 2003), and it had been shown that infusions of human CRP can protect mice against TNF-α-induced hepatotoxicity (Van Molle *et al.*, 1999; Mee-Ree *et al.*, 2000). We therefore investigated whether CRPtg resist lethal experimentally-induced apoptosis. In the first instance mice received i.v. $1 \times 10^7$ to $1 \times 10^9$ plaque-forming-units (pfu) of an adenovirus (Ad) vector that expresses TNF-related apoptosis-inducing ligand (TRAIL) receptor type 2 (R2) upon exposure to tetracycline (Ichikawa *et al.*, 2001). We found that in CRPtg the serum level of mouse SAP and human CRP

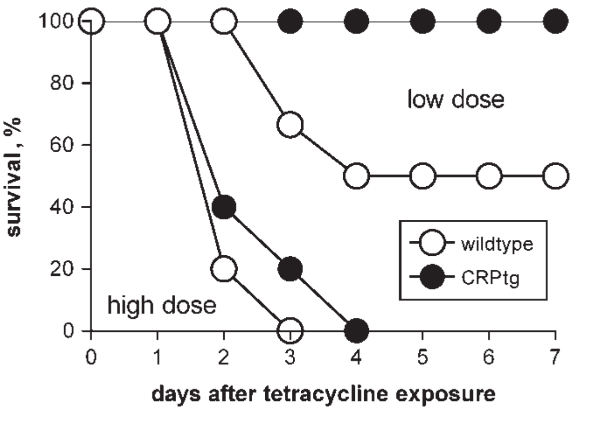

FIGURE 1 CRPtg mice resist lethal apoptosis. On day -3 wildtype and littermate CRPtg mice (10 mice per group) received i.v. $1 \times 10^7$ pfu (low dose) or $1 \times 10^9$ pfu (high dose) of adenovirus vector engineered to express TRAIL-R2 upon exposure to tetracycline (see accompanying text). Expression of TRAIL-R2 induces fulminant apoptosis. At both doses tested, CRPtg had improved survival.

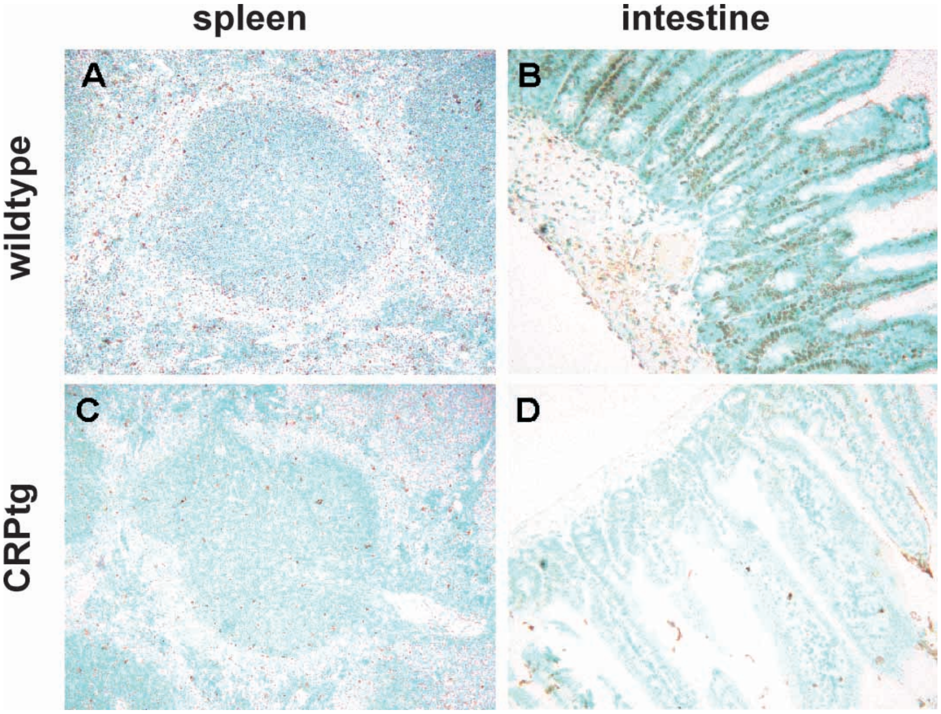

FIGURE 2 Apoptosis induced by infusion of anti-Fas mAb Jo2 is reduced in CRPtg. Spleens and intestines were collected from wildtype C57BL/6 mice (A and B) and congenic CRPtg (C and D) 4 h after injection of 0.344 μg/g Jo2. TUNEL staining was performed to visualize apoptotic cells (brown spots).

decreased concomitant with tetracycline-induced expression of Ad/TRAIL (data not shown), a finding consistent with net depletion of SAP and CRP coincident with induction of apoptosis. Presumably, this depletion is due to participation of SAP and CRP in clearance of apoptotic bodies. CRPtg ultimately had improved survival (Fig. 1). In the second instance, systemic apoptosis was induced in mice by infusion of the anti-Fas antibody Jo2. Mice were injected intravenously with 0.344 μg Jo2/g body weight, and then blood was collected hourly. After 4 h the major organs of mice were removed and fixed in buffered formalin. These were embedded in paraffin and tissue sections were cut and processed to enumerate the number of cells undergoing apoptosis (TUNEL staining). In the spleens of Jo2-injected CRPtg (Fig. 2C) the number of apoptotic cells was reduced substantially compared to spleens from wildtype mice (Fig. 2A). In the columnar epithelium of the intestine, Jo2 induced substantially more apoptosis but this too was greatly reduced in CRPtg (compare Fig. 2, panels D and B). Both observations are preliminary, but they are in agreement with the expectation that CRP contributes to clearance of apoptotic cells.

### CRP-MEDIATED PROTECTION AGAINST AUTOIMMUNITY: A HYPOTHESIS

It is increasingly clear that human CRP protects CRPtg mice against autoimmune disease. Despite the unavoidable caveats (a transgenic mouse is not a human, EAE is not MS, the disease in BW is not SLE, etc.) there is still good reason to believe that CRP has the capacity to protect people with autoimmune disease. At the very least, CRP's antimicrobial properties (Szalai *et al.*, 1995; 2000) should protect rheumatic patients from deleterious infection. We propose that the type and strength of CRP-mediated protection against autoimmune disease varies across a spectrum due to changes in the concentration and glycosylation state of circulating CRP (Fig. 3). This likely determines the exact nature and strength of CRP interaction with apoptotic and

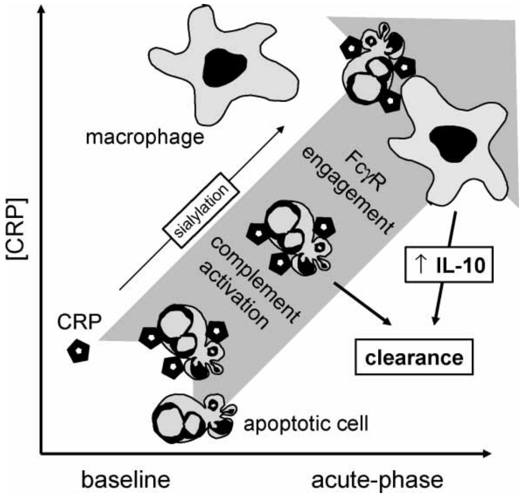

FIGURE 3 Hypothesized relationship between the concentration and glycosylation state of circulating CRP and complement and FcγR mediated clearance of apoptotic cells in autoimmune disease. See accompanying text for details.

effector cells. Under baseline conditions perhaps in concert with complement, CRP could maintain a non-autoimmune state by promoting the "silent" disposal of apoptotic cells. During inflammation perhaps in concert with FcγR, glycosylated CRP could support immune clearance of opsonized targets by monocytes, which secrete IL-10 and dampen inflammation.

### *References*

Abernathy, T.J. and Avery, O.T. (1941) "The occurrence during acute infections of a protein not normally present in the blood. I. Distribution of the reactive protein in patient's sera and the effect of calcium on the flocculation reaction with C-polysaccharide of pneumococcus", *J. Exp. Med.* **73**, 173–182.

Agrawal, A. and Volanakis, J.E. (1994) "Probing the C1q-binding site on human C-reactive protein by site-directed mutagenesis", *J. Immunol.* **152**, 5404–5410.

Agrawal, A., Xu, Y., Ansardi, D., Macon, K.J. and Volanakis, J.E. (1992) "Probing the phosphocholine-binding site of human C-reactive protein by site-directed mutagenesis", *J. Biol. Chem.* **267**, 25352–25358.

Agrawal, A., Lee, S., Carson, M., Narayana, S.V.L., Greenhough, T.J. and Volanakis, J.E. (1997) "Site-directed mutagenesis of the phosphocholine-binding site of human C-reactive protein: role of $Thr^{76}$ and $Trp^{67}$", *J. Immunol.* **158**, 345–350.

Agrawal, A., Shrive, A.K., Greenhough, T.J. and Volanakis, J.E. (2001) "Topology and structure of the C1q-binding site on C-reactive protein", *J. Immunol.* **166**, 3998–4004.

Anderson, J.K., Stroud, R.M. and Volanakis, J.E. (1978) "Studies on the binding specificity of human C-reactive protein for phosphorylcholine", *Fed. Proc.* **37**, 1495.

Ballou, S.P. and Kushner, I. (1992) "C-reactive protein and the acute phase response", *Adv. Intern. Med.* **37**, 313–336.

Berman, S., Gerwurz, H. and Mold, C. (1986) "Binding of C-reactive protein to nucleated cells leads to complement activation without cell lysis", *J. Immunol.* **136**, 1354–1359.

Bharadwaj, D., Stein, M.P., Volzer, M., Mold, C. and Du Clos, T.W. (1999) "The major receptor for C-reactive protein on leukocytes is Fcγ receptor II", *J. Exp. Med.* **190**, 585–590.

Bickerstaff, M.C., Botto, M., Hutchinson, W.L., Herbert, J., Tennent, G.A., Bybee, A., Mitchell, D.A., Cook, H.T., Butler, P.J., Walport, M.J. and Pepys, M.B. (1999) "Serum amyloid P component controls chromatin degradation and prevents antinuclear autoimmunity", *Nat. Med.* **5**, 694–697.

Bodmer, B. and Siboo, R. (1977) "Isolation of mouse C-reactive protein from liver and serum", *J. Immunol.* **118**, 1086–1089.

Burlingame, R.W., Volzer, M.A., Harris, J. and Du Clos, T.W. (1996) "The effects of acute phase proteins on clearance of chromatin from the circulation of normal mice", *J. Immunol.* **156**, 4783–4788.

Ciliberto, G., Arcone, R., Wagner, E.F. and Rüther, U. (1987) "Inducible and tissue-specific expression of human C-reactive protein in transgenic mice", *EMBO J.* **6**, 4017–4022.

Das, T., Sen, A., Kempf, T., Pramanik, S.R., Mandal, C. and Mandal, C. (2003) "Induction of glycosylation in human C-reactive protein under different pathological conditions", *Biochem. J.* **373**, 345–355.

Du Clos, T.W. (1989) "C-reactive protein reacts with U1 small nuclear ribonucleoproteins", *J. Immunol.* **143**, 2553–2559.

Du Clos, T.W., Zlock, L.T. and Rubin, R.L. (1988) "Analysis of the binding of C-reactive protein to histones and chromatin", *J. Immunol.* **141**, 4266–4270.

Du Clos, T.W., Zlock, L.T., Hicks, P.S. and Mold, C. (1994) "Decreased autoantibody levels and enhanced survival of (NZB × NZW)$F_1$ mice treated with C-reactive protein", *Clin. Immunol. Immunopathol.* **70**, 22–27.

Emsley, J., White, H.E., O'Hara, B.P., Olivia, G., Srinivasan, N., *et al.* (1994) "Structure of pentameric human serum amyloid P component", *Nature* **367**, 338–345.

Fischer, W. (2000) "Pneumococcal lipoteichoic and teichoic acids", In: Tomasz, A., ed., *Streptococcus pneumoniae*: Molecular Biology and Mechanisms of Disease (Mary Ann Liebert Inc., New York), pp 155–178.

Gabay, C., Roux-Lombard, P., de Moerloose, P., Dayer, J.M., Wischer, T. and Guerne, P.A. (1993) "Absence of correlation between interleukin 6 and C-reactive protein blood levels in systemic lupus erythematosus compared with rheumatoid arthritis", *J. Rheumatol.* **20**, 815–821.

Gershov, D., Kim, S., Brot, N. and Elkon, K.B. (2000) "C-reactive protein binds to apoptotic cells, protects the cells from assembly of the terminal complement components, and sustains an antiinflammatory innate immune response: implications for systemic autoimmunity", *J. Exp. Med.* **192**, 1353–1363.

Gerwurz, H., Zhang, X.H. and Lint, T.F. (1995) "Structure and function of the pentraxins", *Curr. Opin. Immunol.* **7**, 54–64.

Hind, C.R.K., Collin, P.M., Baltz, M.L. and Pepys, M.B. (1985) "Human serum amyloid P component, a circulating lectin with specificity for the cyclic 4,6-pyruvate acetal of galactose", *Biochem. J.* **225**, 107–111.

Hundt, M., Zielinska-Skowronek, M. and Schmidt, R.E. (2001) "Lack of specific receptors for C-reactive protein on white blood cells", *Eur. J. Immunol.* **31**, 3475–3483.

Ichikawa, K., Liu, W., Zhao, L., Wang, Z., Liu, D., Ohtsuka, T., Zang, H., Mountz, J.D., Koopman, W.J., Kimberly, R.P. and Zhou, T. (2001) "Tumoricidal activity of a novel anti-human DR5 monoclonal antibody without hepatocytes toxicity", *Nat. Med.* **7**, 954–960.

Jefferis, R. and Lund, J. (2002) "Interaction sites on human IgG-Fc for FcγR: current models", *Immunol. Lett.* **82**, 57–65.

Klein, L., Klein, T., Rüther, U. and Kyewski, B. (1998) "CD4 T cell tolerance to human C-reactive protein, an inducible serum protein, is mediated by medullary thymic epithelium", *J. Exp. Med.* **188**, 5–16.

Kushner, I. (1982) "The phenomenon of the acute phase response", *Ann. N Y Acad. Sci.* **389**, 39–48.

Kushner, I. and Mackiewicz, A. (1993) "The acute phases response: an overview", In: Mackiewicz, A., Kushner, I. and Baumann, H., eds, Acute Phase Proteins. Molecular Biology, Biochemistry, and Clincial Applications (CRC Press, Boca Raton), pp 3–20.

Lee, R.T., Takagahara, I. and Lee, Y.C. (2002) "Mapping the binding areas of human C-reactive protein for phosphorylcholine and polycationic compounds", *J. Biol. Chem.* **277**, 225–232.

Marnell, L.L., Mold, C., Volzer, M.A., Burlingame, R.W. and Du Clos, T.W. (1995) "C-reactive protein binds to FcγRI in transfected COS cells", *J. Immunol.* **155**, 2185–2193.

Mee-Ree, C., Park, B.H., Kim, J.S., Rho, H.W., Park, J.W. and Kim, H.R. (2000) "Protective effect of C-reactive protein against the lethality induced by *Vibrio vulnificus* lipopolysaccharide", *Microbiol. Immunol.* **44**, 335–340.

Minota, S., Morino, N., Sakurai, H., Yamada, A. and Yazaki, Y. (1993) "Interrelationship between autoepitope, DNA-binding domain, and CRP-binding domain on a histone H1 molecule", *Clin. Immunol. Immunopathol.* **66**, 269–271.

Mold, C., Gurule, C., Otero, D. and Du Clos, T.W. (1996) "Complement-dependent binding of C-reactive protein complexes to human erythrocyte CR1", *Clin. Immunol. Immunopathol.* **81**, 153–160.

Mold, C., Gewurz, H. and Du Clos, T.W. (1999) "Regulation of complement activation by C-reactive protein", *Immunopharmacology* **42**, 23–30.

Mold, C., Gresham, H.D. and Du Clos, T.W. (2001) "Serum amyloid P component and C-reactive protein mediate phagocytosis through murine FcγRs", *J. Immunol.* **166**, 1200–1205.

Morrow, D.A. and Ridker, P.M. (2000) "C-reactive protein, inflammation, and coronary risk", *Med. Clin. N. Am.* **84**, 149–162.

Osmand, A.P., Friedenson, B., Gewurz, H., Painter, R.H., Hofmann, T. and Shelton, E. (1977) "Characterization of C-reactive protein and the complement subcomponent C1t as homologous proteins displaying cyclic pentameric symmetry (pentraxins)", *Proc. Natl Acad. Sci. USA* **74**, 739–743.

Pepys, M.B. and Baltz, M.I. (1983) "Acute phase proteins with special reference to C-reactive protein and related proteins (pentraxins) and serum amyloid A protein", *Adv. Immunol.* **34**, 141–211.

Pepys, M.B., Baltz, M.L., Gomer, K., Davies, A.J.S. and Doenhoff, M. (1979) "Serum amyloid P component is an acute-phase reactant in the mouse", *Nature* **278**, 259–261.

Pepys, M.B., Lanham, J.G. and DeBeer, F.C. (1982) "C-reactive protein in SLE", *Clin. Rheum. Dis.* **8**, 91–103.

Pereira, D.A., Silva, J.A., Elkon, K.B. and Hughes, G.R.V. (1980) "C-reactive protein levels in systemic lupus erythematosus: a classification criterion?", *Arthritis Rheum.* **23**, 770–771.

Rekvig, O.P., Andreassen, K. and Moens, U. (1998) "Antibodies to DNA—towards an understanding of their origin and pathophysiological impact in systemic lupus erythematosus", *Scand. J. Rheumatol.* **27**, 1–6.

Robey, F.A., Jones, K.D. and Steinberg, A.D. (1985) "C-reactive protein mediates the solubilization of nuclear DNA by complement *in vitro*", *J. Exp. Med.* **161**, 1344–1356.

Schwalbe, R.A., Dahlbäck, B., Coe, J.E. and Nelsestuen, G.L. (1992) "Pentraxin family of proteins interact specifically with phosphorylcholine and/or phosphorylethanolamine", *Biochemistry* **31**, 4907–4915.

Shrive, A.K., Cheetham, G.M.T., Holden, D., Myles, D.A.A., Turnell, W.G., *et al.* (1996) "Three dimensional structure of human C-reactive protein", *Nat. Struct. Biol.* **3**, 346–354.

Stein, M.P., Mold, C. and Du Clos, T.W. (2000) "C-reactive protein binding to murine leukocytes requires Fcγ receptors", *J. Immunol.* **164**, 1514–1520.

Szalai, A.J., Briles, D.E. and Volanakis, J.E. (1995) "Human C-reactive protein is protective against fatal *Streptococcus pneumoniae* infection in transgenic mice", *J. Immunol.* **155**, 2557–2563.

Szalai, A.J., VanCott, J.L., McGhee, J.R., Volanakis, J.E. and Benjamin, W.H., Jr. (2000) "Human C-reactive protein is protective against fatal *Salmonella enterica* serovar typhimurium infection in transgenic mice", *Infect. Immun.* **68**, 5652–5656.

Szalai, A.J., Nataf, S., Hu, X.Z. and Barnum, S.R. (2002) "Experimental allergic encephalomyelitis is inhibited in trangenic mice expressing C-reactive protein", *J. Immunol.* **168**, 5792–5797.

Szalai, A.J., Weaver, C.T., McCrory, M.A., van Ginkel, F.W., Reiman, R.M., Marion, T.N. and Volanakis, J.E. (2003) "Delayed lupus onset in $(NZB \times NZW)_{F1}$ mice expressing a human C-reactive protein transgene", *Arthritis Rheum.* **48**, 1602–1611.

Theofilopoulos, A.N. and Dixon, F.J. (1989) "Experimental murine systemic lupus erythematosus", In: Swanson, J. and Chengerman, K., eds, Animal Models of Disease (US Deptartment of Agriculture, Washington, DC), pp 121–202.

Tomasz, A. (1967) "Choline in the cell wall of a bacterium: novel type of polymer-linked choline in pneumococcus", *Science* **157**, 694–697.

Van Molle, W., Denecker, G., Rodriguez, I., Brouckaert, P., Vandenabeele, P. and Libert, C. (1999) "Activation of caspases in lethal experimental hepatitis and prevention by acute phase proteins", *J. Immunol.* **163**, 235–5241.

Volanakis, J.E. (1982a) "Complement activation by C-reactive protein complexes", *Ann. N Y Acad. Sci.* **389**, 235–250.

Volanakis, J.E. (1982b) "Complement-induced solubilization of C-reactive protein-pneumococcal C-polysaccharide precipitates: evidence for covalent binding of complement proteins to C-reactive protein and pneumococcal C-polysaccharide", *J. Immunol.* **128**, 2745–2750.

Volanakis, J.E. (1982c) "Complement activation by C-reactive protein complexes", *Ann. N Y Acad. Sci.* **389**, 235–250.

Volanakis, J.E. and Kaplan, M.H. (1971) "Specificity of C-reactive protein for choline phosphate residues of pneumococcal C-polysaccharide", *Proc. Soc. Exp. Biol. Med.* **136**, 612–661.

Volanakis, J.E. and Narkates, A.J. (1983) "Binding of human C4 to C-reactive protein-pneumococcal C-polysaccharide complexes during activation of the classical complement pathway", *Mol. Immunol.* **20**, 1201–1207.

Vyse, T.J. and Kotzin, B.L. (1998) "Genetic susceptibility to systemic lupus erythematosus", *Annu. Rev. Immunol.* **16**, 261–292.

Whitehead, A.S., Zahedi, K., Rits, M., Mortensen, R.F. and Lelias, J.M. (1990) "Mouse C-reactive protein: generation of complementary DNA clones, structural analysis, and induction of messenger RNA during inflammation", *Biochem. J.* **266**, 283–290.

Wright, A. and Morrison, S.L. (1997) "Effect of glycosylation on antibody function: implications for genetic engineering", *Trends Biotechnol.* **15**, 26–32.

Xu, Y., Szalai, A.J., Zhou, T., Zinn, K.R., Chaudhuri, T.R., Li, X., Koopman, W.J. and Kimberly, R.P. (2003) "FcγRs modulate cytotoxicity of anti-Fas antibodies: implications for agonistic antibody-based therapeutics", *J. Immunol.* **171**, 562–568.

Young, B., Gleeson, M. and Cripps, A.W. (1991) "C-reactive protein: a critical review", *Pathology* **23**, 118–124.

